# Preoperative fibrinogen/CRP score predicts survival in upper urothelial tract carcinoma patients undergoing radical curative surgery

**DOI:** 10.1007/s00345-023-04379-y

**Published:** 2023-04-06

**Authors:** Valentina Egger, Georg C. Hutterer, Johannes Mischinger, Maximilian Seles, Renate Pichler, Sebastian Mannweiler, Katharina Huber, Amar Balihodzic, Jasmin Spiegelberg, Thomas Bauernhofer, Sascha Ahyai, Richard Zigeuner, Martin Pichler, Dominik A. Barth

**Affiliations:** 1 Division of Oncology, Department of Internal Medicine, Medical University of Graz, Graz, Austria; 2 Department of Urology, Medical University of Graz, Auenbruggerplatz 29, 8036 Graz, Austria; 3 Department of Urology, Medical University of Innsbruck, Innsbruck, Austria; 4 Institute of Pathology, Medical University of Graz, Graz, Austria

**Keywords:** CRP, Fibrinogen, Survival, Upper tract urothelial carcinoma

## Abstract

**Purpose:**

Upper tract urothelial carcinoma (UTUC) represents an often aggressive malignancy associated with poor prognosis. Therefore, finding reliable prognostic biomarkers in patients undergoing curative surgery for improved risk stratification is crucial. We evaluated the prognostic value of the Fibrinogen/C-reactive protein (FC)-score in a cohort of surgically treated UTUC patients.

**Methods:**

170 patients with radiologically and histologically verified UTUC who underwent radical curative surgery between 1990 and 2020, were included. The FC-score was calculated for each patient, with patients receiving 1 point each if Fibrinogen and/or CRP levels were elevated above the 25th or 75th percentile, respectively. Patients were divided into three subgroups according to their FC-score of 0, 1 or 2 point(s). Kaplan–Meier analysis, uni- and multivariable Cox proportional hazard models were implemented. We determined cancer-specific survival (CSS) as primary endpoint, whereas overall survival (OS) and recurrence-free survival (RFS) were considered secondary endpoints.

**Results:**

High FC-score (2 points) was significantly associated with adverse histological features such as vascular invasion (OR = 4.08, 95%CI 1.18–14.15, *p* = .0027) and tumour necrosis (OR = 6.67, 95%CI 1.35–32.96, *p* = 0.020). Both, uni- and multivariable Cox proportional hazard models showed the FC-score as a significant predictor for CSS (univariable analysis: FC-score = 1: HR = 1.90, 95%CI 0.92–3.93, *p* = 0.085 | FC-score = 2: HR = 2.86, 95%CI 1.22–6.72, *p* = 0.016). Furthermore, in univariable analysis, patients with higher FC-score had significantly shorter OS (FC-score = 1: HR = 1.32, 95%CI 0.70–2.49, *p* = 0.387 | FC-score = 2: HR = 2.19, 95%CI 1.02–4.67, *p* = 0.043). However, this did not prevail in multivariable analysis.

**Conclusion:**

The FC-score represents a novel potential biomarker in patients with UTUC undergoing radical curative surgery.

**Supplementary Information:**

The online version contains supplementary material available at 10.1007/s00345-023-04379-y.

## Introduction

Upper tract urothelial carcinoma (UTUC) accounts for roughly 5–10% of all urothelial carcinomas (UCs), and is thus a relatively rare malignancy [1]. The estimated annual incidence of UTUC is about two cases per 100.000 inhabitants in Western Countries [2, 3]. UTUC is located either in the ureter or twice as often in the pyelocaliceal cavities [4]. UCs of the lower and the upper tract do not only differ in localisation but also in practical, anatomical and molecular aspects [5]. Approximately 67% of UTUCs are muscle invasive at the time of diagnosis compared with 15–25% in bladder cancer [2, 6]. The early invasive behavior of UTUC could be a consequence of the anatomical differences between the bladder and the renal pelvis or the ureter, respectively [7]. The muscle layer of the upper urothelial tract (UUT) is thinner than that one of the bladder [7, 8]. Muscle-invasive UTUCs are usually highly aggressive tumours, which are associated with poor prognosis [2, 7, 9]. 5-year-specific survival is < 50% for locally muscle-invasive UTUCs and < 10% for locally advanced UTUCs, which invade adjacent structures [2, 9, 10].

In clinical practice, well-established prognostic factors such as tumour stage and grade [2] are supplemented by other histological factors, including lymphovascular invasion, concomitant carcinoma in situ, variant histology and tumour necrosis [2]. Systemic inflammation (response) has an important impact on the survival, development and progression of cancer [11]. Thus, several preoperative biomarkers and indices based on various immune cells such as the neutrophil/lymphocyte ratio (NLR) [10] and platelet/lymohocte ratio (PLR) [12] have been investigated previously.

Hypercoagulability also plays a major role in several cancers such as renal cell carcinoma [13] or, breast cancer [14] and has been connected to the promotion of angiogenesis, cancer progression and metastasis formation [15]. In the field of cancer medicine, many studies reported that increased hemostatic biomarkers such as fibrinogen are associated with shorter cancer-specific survival (CSS), recurrence-free survival (RFS), metastasis-free survival (MFS), disease-free survival (DFS), and overall survival (OS) in patients with UTUC or other cancers [9, 12, 16]. Gan et al. studied the prognostic value of the FC-score, a combination of the two preoperative blood-based risk factors fibrinogen and C-reactive protein for patients with hepatocellular carcinoma [17]. This FC-score is easy-to-use and could be simply established in clinical practice [17]. As a consequence, we decided to explore if this FC-score is also suitable to be used as a reliable predictor for survival in patients with UTUC.

## Materials and methods

In our observational cohort study, 256 cancer patients with histologically verified UTUC who underwent curative radical surgery at the Department of Urology, Medical University of Graz, Austria, between September 1990 and April 2020, were included in this study. Patients without measured available preoperative CRP and fibrinogen levels were excluded from the study. Clinico-pathological parameters were retrieved from the electronic database system of our Styrian healthcare trust as well as from paperback archives of the Division of Oncology, Department of Internal Medicine, Medical University of Graz, Austria. Assessed parameters included age, sex, gender, ASA score, CRP and fibrinogen levels, as well as histological tumour characteristics such as tumour grade, T-stage, N-stage, intratumoural necrosis and tumour site.

Cut-off values were empirically chosen for both CRP and fibrinogen levels. Patients with CRP levels higher than the 75th percentile of the distribution in our cohort were considered having high CRP values. Likewise, the 25th percentile was chosen as the cut-off for the dichotomisation of fibrinogen levels. The FC-Index was determined as proposed previously by Gan et al. [17]. Patients received one point each if they had high fibrinogen or CRP levels, respectively, and were subsequently assigned to three prognostic groups according to their FC-score. Dates of death were accurately obtained from the Austrian Social Security System database. This study was approved by the local ethics committee (32–645 ex 19/20) of the Medical University of Graz.

### Statistical analyses

Cancer-specific survival, defined as the time (in years) from curative surgery to cancer-related death, was considered the primary endpoint of our study. Cancer-death was assumed if patients had the recurrent disease (locally or distant) within the follow-up period. Secondary endpoints were OS, defined as the time (in years) from the date of surgery to death of any cause, and RFS, defined as the time (in years) from curative surgery to local or distant recurrence. Follow-up was truncated at 10 years for CSS and OS, and at 5 years for RFS, respectively. The Chi-square test was used to assess for associations of the FC-score with clinico-pathological characteristics and univariable logistic regression models were implemented to assess for the predictive ability of the FC-score for parameters that had a significant difference in the distribution in the previous hypothesis test.

Kaplan–Meier estimates were used to plot survival functions and compared by log-rank tests. Further, uni- and multivariable Cox proportional hazard models were implemented. Besides the FC-score, variables, which were significant in univariable analysis were further implemented in the multivariable model. Hazard ratios (HRs) estimated from the Cox analysis were reported as relative risks with corresponding 95% confidence intervals (CIs). All statistical analyses were performed using Stata for Windows version 16.1 (StataCorp LP, Collage Station, TX, USA). A two-sided *p* < 0.05 was considered statistically significant.

## Results

After the exclusion of 125 patients without available preoperative CRP and/or fibrinogen levels, 170 patients from our cohort were included in the final analyses. There was no significant difference of excluded and included patients in clinicopathological parameters, such as age, sex, pelvic tumour, vascular invasion, T-stage, tumour grade, tumour necrosis and lymph nodes (all *p* > 0.005). Yet, a significantly higher proportion of excluded patients showed multifocal UTUC (18.8% *vs.* 36.0%, *p* = 0.001). Median age at the time of surgery was 72 years [interquartile range (IQR) 62.7–78.4 years] and 100 (58.8%) patients were males. Regarding tumour characteristics, 138 (81.2%) patients had multifocal UTUC and 100 (58.8%) patients had involvement of the renal pelvis. See Suppl. Table 1 for a summary of baseline characteristics, including further clinico-pathological parameters like T-stage, N-stage, histological tumour necrosis, and tumour grading.

Median CRP levels were 3.3 mg/L (IQR 0.9–12.0 mg/L) and median fibrinogen levels were 419 mg/dL (IQR 339–512 mg/dL). For biomarker dichotomisation, we chose an empirical cut-off value at the 75^th^ percentile (12 mg/L) regarding the distribution of CRP and at the 25^th^ percentile (339 mg/dL) for fibrinogen. After calculation of the FC-Index according to high or low CRP and fibrinogen levels (one point each for high levels), 38 (22.3%), 95 (55.9%), and 37 (21.8%) patients had an FC-score of zero, one, or two points.

At baseline, there was a significant difference in FC-scores in patients with vascular invasion (chi-square *p* = 0.032), tumour necrosis (chi-square *p* = 0.041), N-stage (chi-square *p* = 0.023). In logistic regression, an FC-score of 2 points was significantly associated with adverse histological features, such as necrosis (odds ratio [OR] = 6.67, 95%CI 1.35–32.96, *p* = 0.020), and vascular invasion (OR = 4.08, 95%CI 1.18–14.15, *p* = 0.0027), while there was no significant association in patients with an FC-score of 1 point (all *p* > 0.05, data not shown).

There was no significant difference in the distribution of age (≤ 65 vs. > 65 years), sex, multifocal UTUC, pelvic tumour, T-stage, tumour grade depending on FC-scores (all *p* > 0.05, s. Suppl. Table 1).

### Survival outcomes according to FC-score prognostic groups

Both, median CSS and OS were not reached in the overall cohort, whereas the median RFS in the overall cohort was 2.1 years (95%CI 1.29-not reached). During the follow-up period of 10 years, there were 59 cancer-related deaths, while 64 patients died of any cause. Overall, 76 patients had a local or distant recurrence of UTUC.

**Fig. 1:**
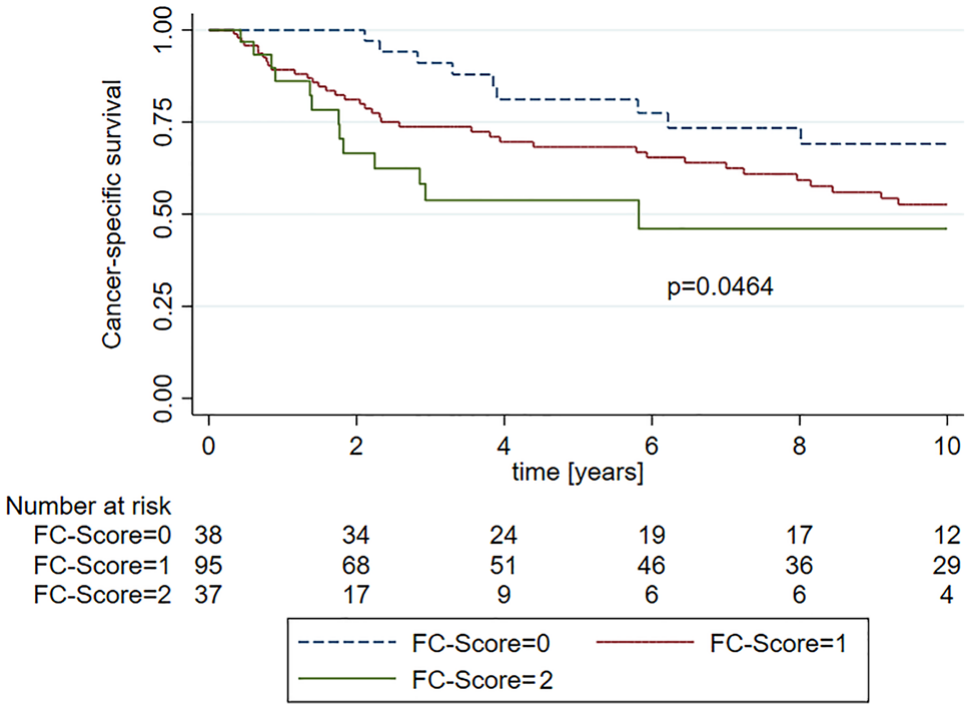
Kaplan–Meier curves showing cancer-specific survival (CSS) for FC-score of 0 points, 1 point and 2 points

**Fig. 2:**
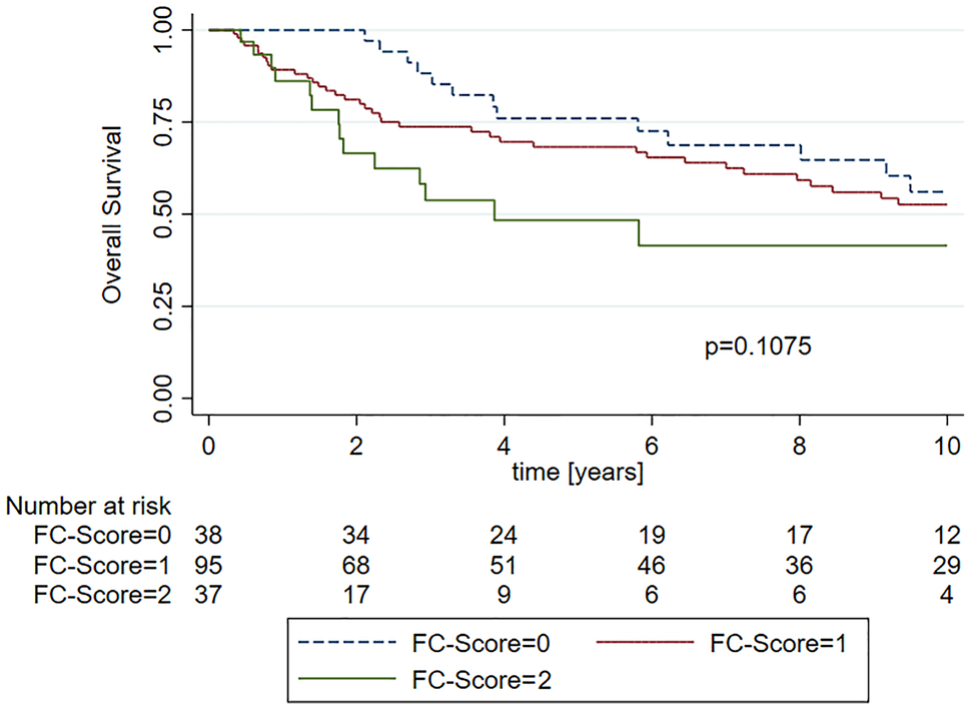
Kaplan–Meier curves showing overall survival (OS) for FC-score of 0 points, 1 point and 2 points

In Kaplan–Meier analysis, patients with higher FC-scores had significantly shorter CSS (Fig. 1, *p* = 0.0464), while there was no significant difference in OS (Fig. 2, *p* = 0.1075) and RFS (Suppl. Figure 1, *p* = 0.9242). However, pairwise comparisons revealed a significant difference in OS between patients with an FC-score of 2 points as compared to patients with 0 points (*p* = 0.0237).

As for our primary endpoint CSS, in univariable analysis, vascular invasion, T-stage, tumour grade, tumour necrosis, and the FC-score (FC-score = 1: HR = 1.90, 95%CI 0.92–3.93, *p* = 0.085 | FC-score = 2: HR = 2.86, 95%CI 1.22–6.72, *p* = 0.016) were significant predictors for CSS. In the multivariable Cox regression model adjusted for vascular invasion, T-stage, tumour grade, and tumour necrosis, the FC-score prevailed a significant predictor of CSS (FC-score = 1: HR = 2.23, 95%CI 1.00–4.97, *p* = 0.049 | FC-score = 2: HR = 1.17, 95%CI 1.00–6.24, *p* = 0.051) [s. Suppl. Table 2].

Regarding OS, patients with high FC-scores had significantly shorter OS as compared to patients with an FC-score of 0 points in the univariable analysis (FC-score = 1: HR = 1.32, 95%CI 0.70–2.49, *p* = 0.387 | FC-score = 2: HR = 2.19, 95%CI 1.02–4.67, *p* = 0.043). However, significance was lost in multivariable analysis adjusted for multifocal UTUC, vascular invasion, T-stage, tumour grade, and tumour necrosis (FC-score = 1: HR = 1.70, 95%CI 0.85–3.41, *p* = 0.132 | FC-score = 2: HR = 2.10, 95%CI 0.93–4.71, *p* = 0.073) [s. Suppl. Table 3]. Considering RFS, there was no significant difference in survival between the three groups in both uni- and multivariable Cox analysis (Suppl. Table 4).

## Discussion

Although several blood-based biomarkers derived from routinely assessed laboratory values have been investigated in UTUC [9, 10, 12], the clinical practice still relies on traditional established prognostic factors, such as tumour stage and grade, which are often determined after radical surgery [2]. Easily-available and non-invasive biomarkers are strongly warranted to assess patient’s risk preoperatively, which could eventually lead to adapted preoperative treatment strategies, such as neoadjuvant systemic therapy, based on patient’s individual risk assumptions.

In this retrospective observational study, we evaluated the FC-score, a combination of the two blood-based markers of inflammation fibrinogen and CRP, in a cohort of 170 UTUC patients who underwent radical curative surgery. The FC-score was a significant and independent predictor of the primary endpoint CSS and, moreover, could successfully differentiate patients into three prognostic groups. Furthermore, we detected signals for the secondary endpoint OS, yet this did not prevail in the multivariable analysis. In addition, a high FC-score was significantly associated with unfavorable histological characteristics, such as necrosis and vascular invasion.

Systemic inflammation is a widely known phenomenon across several cancer entities [18]. Smoldering inflammation promotes tumour development, angiogenesis, metastatic spread and tumour survival [19]. Therefore, inflammation is assumed the seventh hallmark of cancer [11]. A large body of evidence proves CRP, a marker of systemic inflammation routinely used in clinical practice, as a reliable prognostic biomarker across a variety of different treatment settings in various solid cancers [20]. Furthermore, an increase in preoperative CRP has been shown as an adverse prognosticator marker of patient outcomes in UTUC patients undergoing curative resection [21]. Moreover, there are several studies concerning the predictive value of fibrinogen in cancer patients [9, 13, 14, 16], in addition to previous studies in UTUC [9]. Both, CRP as well as fibrinogen play a crucial role in the acute-phase response and are important acute-phase proteins [22, 23]. It is widely known that inflammation triggers the activation of the coagulation system [24] and leads to an increase in prothrombotic and antifibrinolytic factors [23]. Conversely, components of the coagulation system such as fibrinogen or fibrin, respectively, stimulate inflammatory processes and downstream tissue damage. Thus, fibrinogen represents a driver of chronic low-grade inflammation [23]. Moreover, fibrinogen is related to systemic inflammation, such as sepsis, and discussed as a biomarker in the pathogenesis of cardiovascular diseases [25, 26]. An important difference between the two acute-phase proteins CRP and fibrinogen is the difference in half-life times. While CRP has a rather short plasma half-life time of 19 h [27], the half-life time of fibrinogen is significantly longer and lasts around 100 h [28]. Thus, the rationale for combining CRP and fibrinogen in a prognostic inflammation score lies in the inclusion of markers of both, long- and short-term inflammation processes. While preoperative CRP-levels may be heavily influenced by preoperative infections, patients who have both, elevated CRP and elevated fibrinogen levels may have more severe long-lasting and tumour-related inflammatory states, leading to higher FC-scores and worse survival outcomes.

To the best of our knowledge, this is the first study, which explores the FC-score in a cohort of UTUC patients undergoing radical curative surgery. FC-score was previously introduced as a preoperative prognostic biomarker in esophageal squamous cell carcinoma, hepatocellular carcinoma and glioblastoma, were the FC-score was independently associated with OS [17, 29, 30]. Moreover, Tian et al. [30] demonstrated that a high FC-score of 1 or 2 is significantly associated with shortened DFS rates in patients with esophageal squamous cell carcinoma. Gan et al. [17] found that the FC-score seems to be a credible predictor for RFS in patients with hepatocellular carcinoma. Furthermore, Gan et al. [17] were the first to show a division into three prognostic groups by the FC-score in patients with hepatocellular carcinoma, which could now also be shown in our present study in a cohort of UTUC patients undergoing curative resection.

Several important limitations of this retrospective observational study have to be mentioned: Due to the small sample size, our study might be underpowered to detect differences in the secondary endpoints and further larger studies are warranted to confirm our results. Second, due to the retrospective single-center study design, selection bias cannot be excluded entirely. Third, we do not have any records about potential preoperative infections of patients, which might influence preoperative CRP or fibrinogen levels. However, since patients were considered fit for surgery this seems unlikely. Fourth, we detected significant differences in the proportion of multifocal UTUC in excluded and included patients, thus a potential selection bias based on clinico-pathological parameters may not be ruled out and further larger studies are needed.

In conclusion, our results indicate that the FC-score, a combination of the routinely available markers of inflammation fibrinogen and CRP may represent a novel potential biomarker for survival in patients with UTUC undergoing radical surgery.

## Supplementary Information

Below is the link to the electronic supplementary material.

Supplementary file1 Supplementary Fig. 1 Kaplan–Meier curves showing recurrence-free survival (RFS) for FC-score of 0 points, 1 point and 2 points (PDF 60 kb)

Supplementary file2 (DOCX 13 kb)

Supplementary file3 (DOCX 14 kb)

Supplementary file4 (DOCX 14 kb)

Supplementary file5 (DOCX 14 kb)

## Data Availability

The datasets generated and analysed during the current study are available from the corresponding author on reasonable request.
