# Supplementary figures and images for: Preoperative fibrinogen/CRP score predicts survival in upper urothelial tract carcinoma patients undergoing radical curative surgery
Source: World J Urol. 2023 Apr 6;41(5):1359–64. doi: 10.1007/s00345-023-04379-y (PMC10188385; doi:10.1007/s00345-023-04379-y)

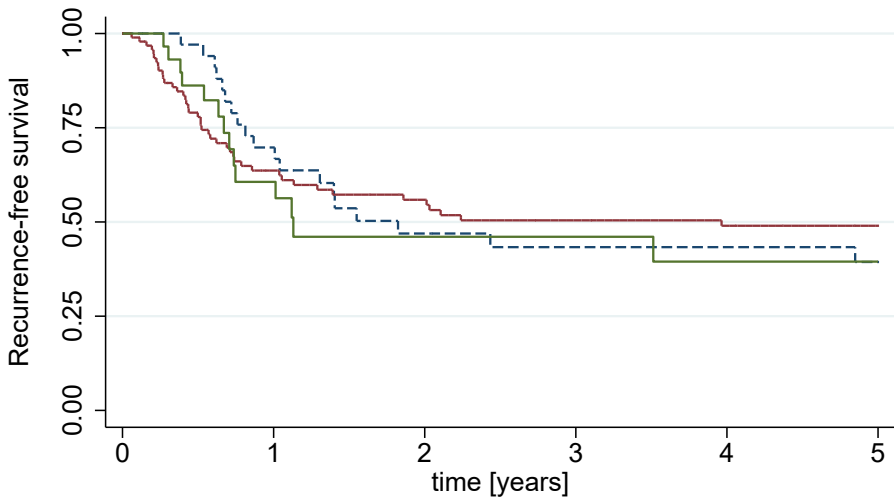

Number at risk

|            |    |    |    |    |    |    |
|------------|----|----|----|----|----|----|
| FC-Score=0 | 38 | 23 | 14 | 12 | 11 | 10 |
| FC-Score=1 | 95 | 50 | 41 | 36 | 34 | 31 |
| FC-Score=2 | 37 | 14 | 9  | 7  | 6  | 5  |

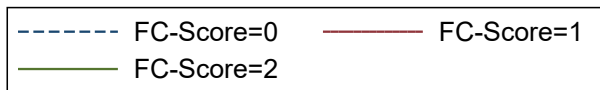

Supplement: Supplementary file 1 — Supplementary file1 Supplementary Fig. 1 Kaplan–Meier curves showing recurrence-free survival (RFS) for FC-score of 0 points, 1 point and 2 points (PDF 60 kb) [file 345_2023_4379_MOESM1_ESM.pdf]
