# Supplementary material for: Preoperative fibrinogen/CRP score predicts survival in upper urothelial tract carcinoma patients undergoing radical curative surgery
Source: World J Urol. 2023 Apr 6;41(5):1359–64. doi: 10.1007/s00345-023-04379-y (PMC10188385; doi:10.1007/s00345-023-04379-y)
Supplement: Supplementary file 2 — Supplementary file2 (DOCX 13 kb) [file 345_2023_4379_MOESM2_ESM.docx]

**Suppl. Table 1: Descriptive characteristics (Summary table) of the study population.**

|  |  | **Overall cohort (n=170)** | | |
| --- | --- | --- | --- | --- |
|  |  | **n (% missing)** | **summary measure** | ***p*-value** |
| **Demographic variables** |  |  |  |  |
| sex |  | 170 (0%) |  | 0.180 |
|  | female |  | 70 (41.18%) |  |
|  | male |  | 100 (58.82%) |  |
| age (yrs.) |  | 170 (0%) |  | 0.094 |
|  | ≤ 65 |  | 52 (30.59%) |  |
|  | > 65 |  | 118 (69.41%) |  |
| **Tumour variables** |  |  |  |  |
| tumour stage |  | 167 (1.76%) |  | 0.124 |
|  | Ta + T1 |  | 79 (46.47%) |  |
|  | T2 - T4 |  | 88 (51.76%) |  |
| tumour grade |  | 170 (0%) |  | 0.137 |
|  | G1 + G2 |  | 91 (53.53%) |  |
|  | G3 + G4 |  | 79 (46.47%) |  |
| multifocal UTUC |  | 170 (0%) |  | 0.059 |
|  | no |  | 138 (81.18%) |  |
|  | yes |  | 32 (18.82%) |  |
| pelvic tumour |  | 170 (0%) |  | 0.278 |
|  | no |  | 70 (41.18 %) |  |
|  | yes |  | 100 (58.82%) |  |
| necrosis |  | 169 (0.59%) |  | 0.041 |
|  | no |  | 139 (81.76%) |  |
|  | yes |  | 30 (18.24%) |  |
| vascular invasion |  | 170 (0%) |  | 0.032 |
|  | no |  | 139 (81.76%) |  |
|  | yes |  | 31 (18.24%) |  |
| nodes |  | 170 (0%) |  | 0.023 |
|  | N0 + NX |  | 159 (93.53%) |  |
|  | N1 - N3 |  | 11 (6.47%) |  |
| **FC-SCORE** |  | 170 (0%) |  |  |
|  | 0 |  | 38 (22.35%) |  |
|  | 1 |  | 95 (55.88%) |  |
|  | 2 |  | 37 (21.76%) |  |
