# Supplementary material for: Preoperative fibrinogen/CRP score predicts survival in upper urothelial tract carcinoma patients undergoing radical curative surgery
Source: World J Urol. 2023 Apr 6;41(5):1359–64. doi: 10.1007/s00345-023-04379-y (PMC10188385; doi:10.1007/s00345-023-04379-y)
Supplement: Supplementary file 3 — Supplementary file3 (DOCX 14 kb) [file 345_2023_4379_MOESM3_ESM.docx]

**Suppl. Table 2: Uni- and multivariable Cox regression models regarding CSS.** HR - hazard ratio, CI - confidence interval

| **Variable** | **Univariable analysis** | | **Multivariable analysis** | |
| --- | --- | --- | --- | --- |
|  | **HR (95% CI)** | ***p*-value** | **HR (95% CI)** | ***p*-value** |
| **Sex**  Male  Female | 1 (reference)  1.256 (0.751-2.101) | 0.385 |  |  |
| **Age (yrs.)**  ≤ 65  > 65 | 1 (reference)  1.429 (0.804-2.542) | 0.224 |  |  |
| **Multifocal**  No  Yes | 1 (reference)  1.513 (0.841-2.720) | 0.167 |  |  |
| **Pelvic tumour**  No  Yes | 1 (reference)  1.280 (0.758-2.161) | 0.355 |  |  |
| **Vascular invasion**  No  Yes | 1 (reference)  8.384 (4.712-14.918) | **<0.001** | 1 (reference)  6.464 (3.184-13.123) | **<0.001** |
| **Tumour stage**  Ta + T1  T2 - T4 | 1 (reference)  2.796 (1.612-4.847) | **<0.001** | 1 (reference)  1.648 (0.864-3.143) | 0.130 |
| **Tumour grade**  G1 + G2  G3 + G4 | 1 (reference)  2.559 (1.517-4.316) | **<0.001** | 1 (reference)  1.689 (0.938-3.042) | 0.081 |
| **Nodes**  N0 + NX  N1 – N3 | 1 (reference)  2.549 (0.914-7.109) | 0.074 |  |  |
| **Tumour necrosis**  No  yes | 1 (reference)  3.413 (1.897-6.143) | **<0.001** | 1 (reference)  0.994 (0.491-2.014) | 0.987 |
| **FC-SCORE**  0  1  2 | 1 (reference)  1.898 (0.916-3.934)  2.861 (1.219-6.720) | 0.085  **0.016** | 1 (reference)  2.233 (1.003-4.970)  2.494 (0.997-6.239) | **0.049**  0.051 |
