# Supplementary material for: Preoperative fibrinogen/CRP score predicts survival in upper urothelial tract carcinoma patients undergoing radical curative surgery
Source: World J Urol. 2023 Apr 6;41(5):1359–64. doi: 10.1007/s00345-023-04379-y (PMC10188385; doi:10.1007/s00345-023-04379-y)
Supplement: Supplementary file 4 — Supplementary file4 (DOCX 14 kb) [file 345_2023_4379_MOESM4_ESM.docx]

**Suppl. Table 3: Uni- and multivariable Cox regression models regarding OS.** HR - hazard ratio, CI - confidence interval

| **Variable** | **Univariable analysis** | | **Multivariable analysis** | |
| --- | --- | --- | --- | --- |
|  | **HR (95% CI)** | ***p*-value** | **HR (95% CI)** | ***p*-value** |
| **Sex**  Male  Female | 1 (reference)  1.168 (0.711-1.919) | 0.539 |  |  |
| **Age (yrs.)**  ≤ 65  > 65 | 1 (reference)  1.499 (0.859-2.616) | 0.154 |  |  |
| **Multifocal**  No  Yes | 1 (reference)  2.029 (1.195-3.445) | **0.009** | 1 (reference)  2.325 (1.268-4.262) | **0.006** |
| **Pelvic tumour**  No  Yes | 1 (reference)  1.277 (0.772-2.110) | 0.341 |  |  |
| **Vascular invasion**  No  Yes | 1 (reference)  7.747 (4.415-13.594) | **<0.001** | 1 (reference)  6.930 (3.485-13.780) | **<0.001** |
| **Tumour stage**  Ta + T1  T2 - T4 | 1 (reference)  2.236 (1.342-3.725) | **0.002** | 1 (reference)  1.425 (0.776-2.619) | 0.254 |
| **Tumour grade**  G1 + G2  G3 + G4 | 1 (reference)  2.435 (1.478-4.012) | **<0.001** | 1 (reference)  1.671 (0.944-2.957) | 0.078 |
| **Nodes**  N0 + NX  N1 – N3 | 1 (reference)  2.416 (0.869-6.715) | 0.091 |  |  |
| **Tumor necrosis**  No  yes | 1 (reference)  3.144 (1.762-5.608) | **<0.001** | 1 (reference)  0.736 (0.342-1.581) | 0.432 |
| **FC-SCORE**  0  1  2 | 1 (reference)  1.322 (0.702-2.488)  2.188 (1.024-4.674) | 0.387  **0.043** | 1 (reference)  1.704 (0.852-3.408)  2.098 (0.934-4.715) | 0.132  0.073 |
