# Supplementary material for: Preoperative fibrinogen/CRP score predicts survival in upper urothelial tract carcinoma patients undergoing radical curative surgery
Source: World J Urol. 2023 Apr 6;41(5):1359–64. doi: 10.1007/s00345-023-04379-y (PMC10188385; doi:10.1007/s00345-023-04379-y)
Supplement: Supplementary file 5 — Supplementary file5 (DOCX 14 kb) [file 345_2023_4379_MOESM5_ESM.docx]

**Suppl. Table 4: Uni- and multivariable Cox regression models regarding RFS.** HR - hazard ratio, CI - confidence interval

| **Variable** | **Univariable analysis** | | **Multivariable analysis** | |
| --- | --- | --- | --- | --- |
|  | **HR (95% CI)** | ***p*-value** | **HR (95% CI)** | ***p*-value** |
| **Sex**  Male  Female | 1 (reference)  1.343 (0.853-2.114) | 0.203 |  |  |
| **Age (yrs.)**  ≤ 65  > 65 | 1 (reference)  0.946 (0.586-1.527) | 0.820 |  |  |
| **Multifocal**  No  Yes | 1 (reference)  2.121 (1.306-3.446) | **0.002** | 1 (reference)  1.790 (1.031-3.107) | **0.038** |
| **Pelvic tumour**  No  Yes | 1 (reference)  1.311 (0.825-2.085) | 0.252 |  |  |
| **Vascular invasion**  No  Yes | 1 (reference)  3.785 (2.281-6.279) | **<0.001** | 1 (reference)  3.266 (1.691-6.308) | **<0.001** |
| **Tumour stage**  Ta + T1  T2 - T4 | 1 (reference)  1.709 (1.073-2.722) | **0.024** | 1 (reference)  1.076 (0.601-1.926) | 0.806 |
| **Tumour grade**  G1 + G2  G3 + G4 | 1 (reference)  1.824 (1.159-2.869) | **0.009** | 1 (reference)  1.439 (0.866-2.388) | 0.160 |
| **Nodes**  N0 + NX  N1 – N3 | 1 (reference)  2.197 (0.875-5.519) | 0.094 |  |  |
| **Tumour necrosis**  No  yes | 1 (reference)  2.498 (1.458-4.282) | **0.001** | 1 (reference)  1.027 (0.511-2.063) | 0.940 |
| **FC-SCORE**  0  1  2 | 1 (reference)  0.976 (0.568-1.675)  1.103 (0.552-2.202) | 0.929  0.781 | 1 (reference)  1.005 (0.561-1.799)  0.930 (0.453-1.907) | 0.988  0.843 |
